# HLA-E^⁎^01:03 Allele in Lung Transplant Recipients Correlates with Higher Chronic Lung Allograft Dysfunction Occurrence

**DOI:** 10.1155/2016/1910852

**Published:** 2016-07-17

**Authors:** Julie Di Cristofaro, Mathieu Pelardy, Anderson Loundou, Agnès Basire, Carine Gomez, Jacques Chiaroni, Pascal Thomas, Martine Reynaud-Gaubert, Christophe Picard

**Affiliations:** ^1^CNRS, EFS, ADES UMR 7268, Aix-Marseille Université, 13916 Marseille, France; ^2^Immunogenetics Laboratory, EFS-Alpes Méditerranée, 13005 Marseille, France; ^3^Department of Public Health, EA 3279 Research Unit, Marseille University Hospital, Aix-Marseille University, Marseille, France; ^4^Service de Pneumologie, Hôpital Nord, 13015 Marseille, France; ^5^Service de Chirurgie Thoracique, Hôpital Nord, 13015 Marseille, France

## Abstract

Lung transplantation (LTx) is a valid therapeutic option for selected patients with end-stage lung disease. HLA-E seems to play a major role in the immune response to different viral infections and to affect transplantation outcome, in Hematopoietic Stem Cell Transplantation, for example. Two nonsynonymous alleles, HLA-E^⁎^01:01 and HLA-E^⁎^01:03, have functional differences, involving relative peptide affinity, cell surface expression, and potential lytic activity of NK cells. The aim of this retrospective study was to determine the impact of these two alleles for LTx recipients on anti-HLA alloimmunization risk, overall survival, and chronic rejection (CLAD). HLA-E was genotyped in 119 recipients who underwent LTx from 1998 to 2010 in a single transplantation center. In univariate analysis, both HLA-E homozygous states were associated with impaired overall survival compared to heterozygous HLA-E alleles (*p* = 0.01). In multivariate analysis, HLA-E^⁎^01:03 allele showed increased CLAD occurrence when compared to homozygous HLA-E^⁎^01:01 status (HR: 3.563 (CI 95%, 1.016–12), *p* = 0.047). HLA-E allele did not affect pathogen infection or the production of* de novo* DSA. This retrospective study shows an uninvestigated, deleterious association of HLA-E alleles with LTx and requires verification using a larger cohort.

## 1. Introduction

Lung transplantation (LTx) is a valid therapeutic option for selected patients with end-stage lung disease. Unfortunately, posttransplant prognosis is hampered by the occurrence of chronic lung allograft dysfunction (CLAD) which is highly prevalent and remains the major limitation to long-term survival and functional outcome in LTx compared to other solid-organ transplants [1]. CLAD commonly reflects a bronchiolar obstruction defining a bronchiolitis obliterans syndrome (BOS). Recently, another phenotype of CLAD with a predominant restrictive pattern has been identified and called restrictive allograft syndrome (RAS) [2]. BOS and probably RAS are considered to be a multistep injury remodeling phenomenon targeted by recurrent immunologic events such as acute rejection and the development of* de novo* Donor Specific Antibodies (DSA). Several nonimmunological risk factors have been proposed, although not yet widely accepted as cytomegalovirus pneumonitis, bacterial/fungal/non-CMV viral infections, and persistent neutrophil influx and sequestration. Also, various genetic factors have been identified such as TGF- (Transforming Growth Factor-) B1, Toll-Like Receptor (TLR), and IL- (Interleukin-) 17. However, confirmation was not consistent across all studied cohorts [3].

Recently, we showed that HLA-G genetic polymorphism could be associated with LTx outcome, especially with CLAD occurrence [4]. HLA-G is a nonclassical HLA class I molecule, closely related to HLA-E: the HLA-E molecule also plays a crucial role in inflammatory and adaptive immune responses. HLA-E binds preferentially to the inhibitory CD94/NKG2A and activating CD94/NKG2C (which lacks ITIM motif) receptors, selectively expressed on NK cells and a subset of CTL cells, modulating their cell-mediated activity [5]. Furthermore, HLA-E has also been showed to react with CD8 T cells expressing the conventional T Cell Receptor (TCR), suggesting that HLA-E involvement in the adaptive immune system responses is mediated by T cells [6].

HLA-E mRNA is expressed in most tissues [7], but its cell surface expression appears to be controlled by the binding of a range of different peptides, such as signal peptides derived from classical and nonclassical MHC class I molecules, stress protein peptides, and peptides derived from different pathogens [8, 9]. Thus, physiologically, HLA-E is expressed at the cell surface of endothelial cells, T and B lymphocytes, monocytes, and macrophages [10].

As HLA-G, HLA-E displays limited polymorphism with 21 alleles listed in the IMG/HLA database (release 3.23.0), including 9 proteins. HLA-E^*∗*^01:01 and HLA-E^*∗*^01:03 are the main alleles observed, with similar frequencies (~50%) in different populations [11–14]. They differ at codon 107 (R/G) and encode HLA-ER (E^*∗*^01:01) and HLA-EG (E^*∗*^01:03) proteins. Functional differences between these two isoforms involve relative peptide affinity, cell surface expression, and potential lytic activity of NK cells [15]. Indeed, HLA-E^*∗*^01:03 protein is more thermally stable, exhibits higher cell surface expression, and is a potentially stronger inhibitor of the NK cell's lytic activity compared to HLA-E^*∗*^01:01 [15].

A limited number of studies, with conflicted results, investigated into the influence of HLA-E genotype on transplantation outcomes. Although it is very difficult to show an impact of HLA-E polymorphism in HSCT, because of a limited amount of clinical data (cohort size, patient treatment differences), the majority of studies supported an association between the HLA-E^*∗*^01:03 allele and a lower risk of graft-versus-host disease, decreased mortality, and greater disease-free survival suggesting graft-versus-leukemia (GVL) effect after Hematopoietic Stem Cell Transplantation (HSCT) [16–20].

Control of HLA-E cell surface expression by bacterial or viral infections and particularly human CMV is well established [21]. One study showed that CMV-associated HLA-E-restricted T cell alloreactivity was tightly regulated by NK receptors [22]. Furthermore, a recent study demonstrated that CMV-associated HLA-E-restricted T cells from a kidney transplant recipient recognize and lyse allogenic endothelial cells independently of their CMV status and HLA-E genotype, supporting a potentially detrimental HLA-E alloreactivity [23]. Interestingly, HLA-E molecules expressed in transgenic mice elicited an alloantigenic reaction indistinguishable from classical MHC class I molecules [24]. Finally, natural HLA antibodies directed against HLA-E have been detected in the sera of nonalloimmunized healthy male donors, probably induced by cross-reactive bacterial antigens and/or peptides derived from ingested food or allergens [25].

Since immunosuppressed lung transplant patients are particularly sensitive to infection and HLA sensitization, we speculated that HLA-E genotype could have an influence on the lung protection/allograft rejection balance.

Considering the immunotolerogenic properties of HLA-E, the main objectives of this study were to determine the impact of HLA-E alleles in a cohort of 138 adult LTx recipients on overall survival, disease-free survival (with and without CLAD), and viral and bacterial infection and on HLA sensitization.

## 2. Material and Methods

### 2.1. Study Design and Patient Characteristics

We conducted a retrospective single-center study based on adult patients who underwent LTx at the Marseille Lung Transplant Center between December 1998 and December 2010. Patients who had less than 90-day survival or who were lost to follow-up were excluded. There were 138 adult LTx recipients (mean (SD) age: 39.3 (16.3) years, 64 women and 55 men) eligible for analysis.

Patients received a first lung transplant (26 single LTx; 112 bilateral LTx) for an initial diagnosis of cystic fibrosis (43%), emphysema (25%), pulmonary fibrosis (20%), or another diagnosis (12%). The mean age at transplant procedure was 39.3 (13.3) years. The mean follow-up time was 37.2 months (CI: 10.8–63.2 months). HLA-A, HLA-B, HLA-DR, and HLA-DQ mismatching between donor and recipient were 56%, 82%, 68%, and 53%, respectively.* De novo* post-LTx DSA were detected in 51 recipients (38%) using Luminex single-antigen flow beads. Forty-two, 26, 11, and 11 post-LTx DSA were detected at Month 1, M3, M12, and M24, respectively [3].

### 2.2. Posttransplant Clinical Management

Immunosuppression and prophylaxis: all recipients received a similar standardized immunosuppressive regimen in accordance with our institutional protocols. Induction therapy consisted of intravenous administration of rabbit anti-thymocyte globulins (Pasteur Merieux, Lyon, France) given for the first 3 postoperative days (except when daily lymphocyte count was below 200/mm^3^ and when there were cytomegalovirus (CMV) and/or EBV mismatches, i.e., seronegative recipient and seropositive donor). A high dose of methylprednisolone was additionally administered (6 mg/kg/d Day 1, 2 mg/kg/d Day 2 and Day 3, and 1 mg/kg/d thereafter). The standard triple maintenance immunosuppressive regimen consisted of cyclosporin (adjusted to maintain whole blood trough levels varying between 200 and 250 ng/mL) or tacrolimus after 2003 (adjusted to maintain whole blood trough levels varying between 8 and 12 ng/mL), azathioprine (1 mg/kg/d) or mycophenolate mofetil after 2003 in 5 patients (adjusted to a white blood cell count above 4000 mm^3^), and steroids (prednisone) tapered to 0.25 mg/kg/d over the first 3 months and stopped if possible around 12 months after surgery.

Episodes of acute cellular allograft rejection were treated with intravenous methylprednisolone (5 mg/kg/d for 3 consecutive days) and then rapidly reduced. All CMV-positive recipients were treated with IV ganciclovir switched to oral valganciclovir as soon as possible for prophylaxis over the first 15 days after transplantation. All CMV-mismatched recipients (seronegative recipient and seropositive donor) were treated with IV ganciclovir or oral valganciclovir when available for prophylaxis for the first 3 postoperative months. CMV-negative recipients who received a graft from a negative donor did not receive antiviral prophylaxis. All recipients were screened weekly for CMV infection with a polymerase chain reaction assay and pp65 antigenemia for the first 12 weeks, monthly thereafter, and when clinically indicated.

Pulmonary function tests (PFTs) were routinely conducted at our center on a monthly basis for the first 12 postoperative months, at 2-month intervals in the second year, and at 3-month intervals thereafter. In addition, PFTs were conducted when patients had clinical symptoms or a decline in home spirometry values of at least 10% on 2 consecutive days. Spirometry was measured in a constant volume (830 L) whole body plethysmograph (MasterLab, Jaeger, Wurzburg, Germany) and included measurement of forced vital capacity (FVC), FEV_1_, residual volume (RV), and total lung capacity (TLC). Forced expiratory flow rate between 25% and 75% of FVC (FEF_25–75_) was obtained from the best flow-curve volume. The baseline FEV_1_ value was calculated as the average of the 2 best FEV_1_ values at least 3 weeks apart. Baseline values of TLC and FEV_1_/FVC were defined as the average of the 2 measurements obtained at the same time as the best 2 FEV_1_ measurements.

The diagnosis of CLAD included both BOS and RAS phenotypes. BOS was defined according to the ISHLT guidelines [19]. RAS was defined as an irreversible decline in TLC to <90% of baseline for more than 3 weeks [2].

### 2.3. HLA-E Genotyping

A home-made primer extension method described in Julie et al., 2011, was used to simultaneously analyze 5 SNPs of the HLA-E gene in 119 patients to detect HLA-E^*∗*^01:01, HLA-E^*∗*^01:02, HLA-E^*∗*^01:03, and HLA-E^*∗*^01:04 alleles [11]. Nineteen acid nucleic samples were either lost (10) or degraded (9).

Briefly, the HLA-E multiplex PCR coamplified two PCR fragments (exons 1-2 by forward primer TGGTAGATGGAACCCTCCTTT and reverse primer GTGAATCTGGGACCCGAAG and exon 3 by forward primer GTGGGCGGGACTGACTAAG and reverse primer AGTAGCCCTGTGGACCCTCT) encompassing three and two SNPs, respectively. The multiplex PCR was performed on 200 and 75 ng of genomic DNA, respectively, in a final volume of 25 *μ*L containing 1x PCR buffer, 1.5 mM MgCl_2_, 0.2 mM of each dNTP, and 0.1 units of Taq DNA-polymerase (Invitrogen, Cergy-Pontoise, France) and a defined concentration of each primer. Amplification was carried out as follows: 1 cycle at 95°C for 5 min; 30 cycles at 95°C for 30 s, 63°C for 45 s, and 72°C for 75 s; and 1 cycle at 72°C for 7 min. After control on 2% (w/v) agarose gel, 15 *μ*L of PCR product was incubated with 5 units of shrimp alkaline phosphatase and 1 unit of exonuclease-I (Euromedex, Souffelweyersheim, France) for 1 h at 37°C followed by 15 min at 80°C to remove unincorporated primers and dNTPs.

The second step involved the incorporation of a fluorescent dNTP into extension-primers annealed upstream or downstream to each SNP. This multiplex extension reaction including five forward or reverse extension-primers (48T-AGTGTGGAAATACTTCAAGGAGTG to detect polymorphism C/G in codon 66, 10T-CCGCACAGATTTTCCGAGTGAA to detect polymorphism C/T in codon 77, 25T-CTGCGGACGCTGCG to detect polymorphism C/G in codon 83, 41T-CGCGGAGGAAGCGCC to detect polymorphism A/G in codon 107, and 41T-GCATGTGTCTTCCAGGTAGGCTC to detect polymorphism G/A in codon 157) was performed using the SNaPshot kit (Applied Biosystems, Courtaboeuf, France) according to the manufacturer's protocol in a final volume of 10 *μ*L containing 3 *μ*L of the PCR product, 5 *μ*L of SNaPshot mix, and extension-primers. The reaction was programed as follows: 25 cycles at 95°C for 10 s, 50°C for 5 s, and 60°C for 30 s. The product of the extension reaction (10 *μ*L) was purified with 1 unit of SAP for 1 h at 37°C, followed by 15 min at 80°C for enzyme inactivation.

The fluorescence and size of the extended products were determined by capillary electrophoresis on an ABI PRISM 3130XL genetic analyzer (Applied Biosystems) using POP-7 polymer and a 36 cm capillary array. Capillary electrophoresis was performed according to the manufacturer's protocol.

Data were analyzed using GENEMAPPER v4.0 with specific detection parameters. Using an in-house computer program, output files (.txt) exported from GENEMAPPER 4.0 were automatically formatted into files readable by the “*Phenotype*” application of the GENE[RATE] computer tool package [26].

HLA-G and UTR genotyping data were from [4].

### 2.4. Studied Variables

Variables applied in univariate and multivariable analyses were grouped into four categories:

1. Preoperative donor variables: donor age, gender, CMV status, and classical HLA type.
2. Preoperative and postoperative recipient variables: recipient age, gender, initial diagnosis, HLA type and HLA Donor Specific Antibodies (DSA), CMV status and bacterial infection at M1 and M3, HLA-E, and HLA-G polymorphism.
3. Preoperative donor-recipient matching: age, gender, CMV mismatch, and HLA mismatch.
4. Intraoperative variables: ischemic time and type of procedure (single versus bilateral LTx).

### 2.5. Statistical Analyses

Missing data led to the exclusion of the concerned sample from further analyses. No multiple imputations were used in this study.

HLA-G and HLA-E Global Linkage Disequilibrium and HLA-E frequencies were estimated using an EM algorithm implemented in the GENE[RATE] computer tools. Deviations from Hardy-Weinberg equilibrium (HWE) were tested using a nested likelihood model.

Median values and ranges were used for continuous variables and percentages for categorical variables. For each continuous variable, the study cohort was initially split into quartiles and into two groups at the median.

Analysis using the Kruskal-Wallis test, Fisher's exact test, and Chi-square test was applied to determine clinical significance wherever required, in particular for any relevance between HLA-E alleles and the status of CMV infection.

The primary endpoints of this study were overall survival (OS) and disease-free survival (DFS). OS was defined as the interval between the date of transplantation and last follow-up visit or death. DFS was defined as the time interval from transplantation to the first event, either the diagnosis of CLAD or death without diagnosis of CLAD. The Kaplan-Meier method was used to estimate OS and freedom from CLAD. The log-rank test was used to assess the univariate effects on OS and DFS. For all analyses, *p* < 0.05 was considered statistically significant.

Multivariate analyses were performed using Fine and Gray's proportional hazards regression model.

All analyses were performed using SPSS 15.0 software (SPSS Inc., Chicago, IL) and the cmprsk package (developed by Gray, June 2001) on R2.3.0 software (http://www.R-project.org/).

## 3. Results

### 3.1. Conditions Associated with Overall Survival and Allograft Function

Overall survival of the LTx population was 74% and 68% at 12 and 24 months, respectively (median survival, 7 years). In univariate analysis, the conditions associated with better survival were an initial diagnosis of cystic fibrosis (CF) compared to other indications, HLA class II DSA detected at M3 and/or M12, HLA-G^*∗*^01:04~UTR3 haplotype, the younger age of the recipient, absence of CLAD occurrence, the number of recipient/donor mismatches at the B locus, and HLA class I DSA detected at M3 and/or M12 (Table 1(a)). During the study period, 29 LTx recipients developed CLAD (including 5 patients (17%) with RAS and 24 (83%) with BOS), corresponding to a proportion of 8%, 21%, and 30% of the cohort at 1, 2, and 3 years after LTx, respectively. The major risks for CLAD were HLA class I DSA detected at M3 and/or M12, recipient/donor mismatches at HLA-DR and HLA-DQ loci, the single lung transplant procedure, the non-CF population, and carrying the HLA-G^*∗*^01:04~UTR3 haplotype (Table 1(b)).

### 3.2. HLA-E Allelic Frequencies

A total of 3 HLA-E genotypes were observed in these patients (Table 2). Homozygous HLA-E^*∗*^01:01 was detected in 38/119 (32%) patients, homozygous HLA-E^*∗*^01:03 was detected in 24/119 (20%), and heterozygous HLA-E was detected in 57/119 (48%). HLA-E frequency distribution was in Hardy-Weinberg equilibrium. HLA-E allele and genotype frequencies were also concordant with previously published data showing a relatively equal distribution between HLA-E^*∗*^01:01 and HLA-E^*∗*^01:03 in Western Europe [11]. Analysis of two-locus Global Linkage Disequilibrium (GLD) showed that the HLA-E alleles are not in significant GLD with HLA-G or UTR loci.

### 3.3. Impact of HLA-E Alleles and Genotypes on LTx Outcome

**Figure 1:**
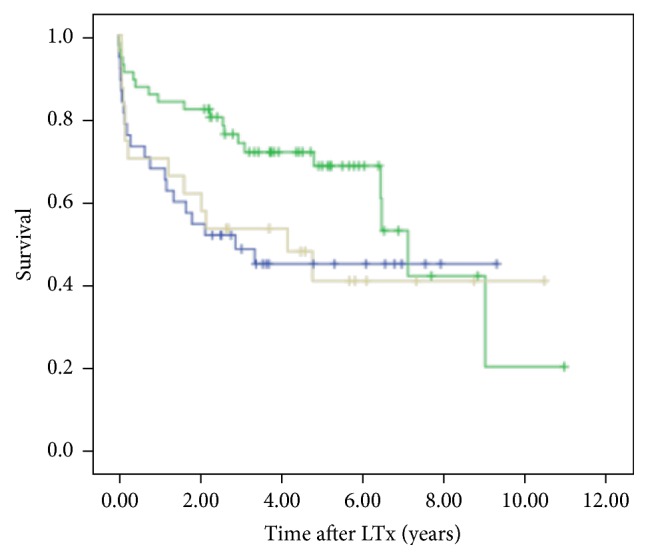
Survival curves in LTx recipients according to the presence of heterozygous HLA-E (in green) compared to homozygous HLA-E (homozygous HLA-E^*∗*^01:01 in light grey, homozygous HLA-E^*∗*^01:03 in blue) (log-rank test, *p* = 0.01).

**Figure 2:**
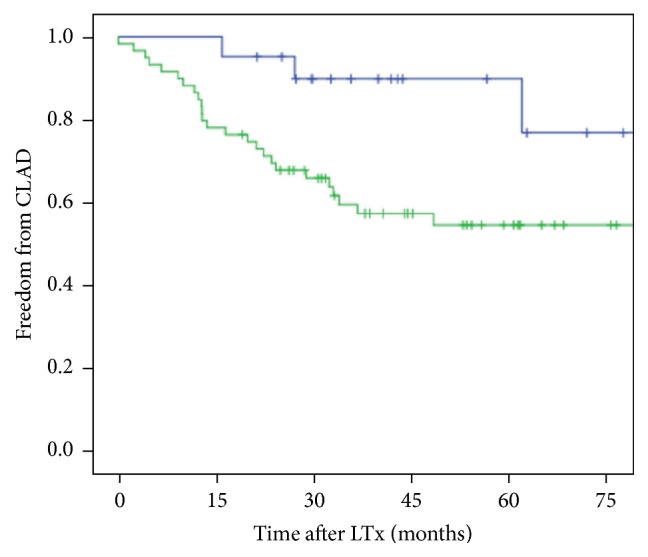
Freedom from CLAD in LTx recipients according to the presence of homozygous HLA-E^*∗*^01:01 (in blue) compared to HLA-E^*∗*^01:03 alleles (in green) (log-rank test, *p* = 0.02).

HLA-E genotype was not associated with patient characteristics (Table 2). HLA-E^*∗*^01:01/01:03 heterozygous state was associated with survival advantage (*p* = 0.01) when compared to HLA-E^*∗*^01:01 or HLA-E^*∗*^01:03 homozygous states. The two-year survival rate was 80% ± 8% for HLA-E heterozygous genotypes and 50% ± 8% and 50% ± 10% for homozygous HLA-E^*∗*^01:01 and homozygous HLA-E^*∗*^01:03 states, respectively (Figure 1). HLA-E^*∗*^01:03 allele was associated with CLAD occurrence (Figure 2). The two-year freedom from CLAD rate was 44% ± 25% for the HLA-E^*∗*^01:03 allele and 73.4% ± 8% for the homozygous HLA-E^*∗*^01:01 state.

Cox proportional regression hazards modeling showed that the main risk factors for CLAD were a noncystic fibrosis initial diagnosis (*p* < 0.001), HLA-E^*∗*^01:03 alleles carriers (*p* = 0.047), and HLA-G^*∗*^01:04~UTR3 carriers (*p* = 0.071). The relative risks of CLAD were 8.612 for emphysema compared to cystic fibrosis patients (CI 95%, 3.13–23.824), 3.563 in recipients carrying HLA-E^*∗*^01:03 allele compared to recipients who did not carry it (CI 95%, 1.016–12), and 3.037 in recipients carrying HLA-G^*∗*^01:04~UTR3 compared to recipients who did not carry it (CI 95%, 0.889–7.412) (Table 3).

None of the HLA-E alleles were significantly associated with the different clinical variables, such as bacterial infection at M1 or M3 and DSA detection at M1 and M3 (Table 2).

## 4. Discussion

This is the first study to show that HLA-E polymorphisms could be implicated in survival and CLAD occurrence in LTx. The two homozygous HLA-E states are associated with worse survival compared to the heterozygous state. These alleles however contribute differentially as HLA-E^*∗*^01:03 allele is correlated to CLAD occurrence in multivariate analysis.

Although the exact pathogenesis of CLAD remains unknown, studies indicate that BOS begins with epithelial injury of the airways due to a variety of factors such as viral infection, autoimmune disease, and alloreactivity response, followed by an inflammatory reaction that leads to obliteration of the airways [3]. HLA-E is an immunomodulatory molecule that can function as both an immune-tolerogenic and immune-activating molecule and plays a dual role in natural and acquired immune responses.

The HLA-E-peptide complex can act as ligand for the CD94/NKG2 receptors expressed on the surface natural killer cells and represents a restriction element for the TCR. Although the two alleles only differ by a single amino acid in alpha 2 domain of the HLA-E heavy chain, HLA-E^*∗*^01:03 is characterized by a stronger affinity for various peptides and a higher thermal stability than HLA-E^*∗*^01:01, inducing its higher cell surface expression in PBMC or other cells [27]. These differences might influence the affinities for the different activator or inhibitor receptors, might induce different intracellular signaling, and might have an impact on the cellular immune response in the context of transplantation [28].

HLA-E has the ability to bind non-self-antigen and self-antigen, among which peptides derived from the leader sequences of classical and nonclassical HLA molecules. HLA-E and HLA-G, both categorized as nonclassical class I HLA, share immunosuppressive properties and immunological cell targets; furthermore, HLA-E has the highest affinity for the HLA-G leader peptide. These observations back quite a close relationship between these molecules, but this coordination remains unclear. For instance, in the context of pregnancy, HLA-E, binding an HLA-G peptide signal, interacts with CD94/NKG2C activating receptors to activate NK cells lysis of trophoblast cells during placental invasion, leading to tissue remodeling [29]. At a genetic level, the low number of coding SNPs in both HLA-E and HLA-G loci could suggest that amino acid modifications have serious functional consequences. However, HLA-G and HLA-E are not in GLD. Furthermore, the impact of HLA-G^*∗*^01:04~UTR3 on CLAD occurrence seems to be independent of the HLA-E^*∗*^01:03 allele; finally, contrary to HLA-G alleles, HLA-E^*∗*^01:03 was not associated with an increase in anti-HLA alloimmunization, suggesting that HLA-E and HLA-G have both differential involvements and pathways. Thus, another hypothesis to explain the low level of coding modification in both HLA-E and HLA-G loci is that they lead to amino acid modifications with little functional consequences but that they are linked to SNPs in the regulatory region involved in quantitative expression.

Considering HLA-E and pathogen infections, HLA-E can bind to different viral peptides derived from CMV, the Epstein-Barr virus, the human immunodeficiency virus, the influenza virus, and Hepatitis C virus. A few studies have linked reactivation of CMV with organ rejection [30]. CMV infection in post-LTx is considered a risk factor for BOS [30, 31]. The CMV immune evasion protein, UL40, when complexed with HLA-E, can modulate NK cell functions via interactions with the CD94-NKG2A receptors, leading to viral evasion [31]. Recently, it has been suggested that latent CMV infection-mediated increase in the proportion of NKG2C+ NK cells may prime NK cell cytotoxicity and could be beneficial in preventing the progression and development of hematologic malignancies characterized by high HLA-E expression [32]. This effect, dependent potentially on HLA-E alleles, may be deleterious for transplantation occurrence.

Furthermore, the UL40-derived sequence can also be immunogenic, eliciting robust CD8+ T cell responses. Recently, CMV UL-40-specific T cells were detected in the peripheral blood of LTx recipients and were significantly associated with allograft dysfunction, such as BOS [33]. These cells were first identified between 6 and 12 months after transplant, a period that coincides with the cessation of antiviral prophylaxis and the highest risks for CMV reactivation, suggesting an antigen-driven expansion restricted preferentially by HLA-E. These cells could lyse a large array of allogenic target cells and directly damage the allograft [34]. In contrast, HLA-E can promote specific HLA-E-restricted CD8+ Treg cells that inhibit antiviral effector CD8+ T cells, diminishing virus control [35].

In this retrospective study, CMV reactivation data were unavailable. Mismatch CMV (D−/R− versus D+/R− or + and D+ or −/R+) were not associated with CLAD occurrence and overall survival (*p* = 0.8 and *p* = 0.7, resp.). None of the homozygous or heterozygous HLA-E alleles with a positive CMV recipient and/or donor showed a statistical difference on survival and CLAD occurrence compared to the same HLA-E alleles with a negative CMV recipient and donor (*p* = 0.8 and *p* = 0.7) (data not shown). The percentage of death (55%) was similar between positive CMV recipient and/or donor and negative CMV donor and recipient, whatever homozygous HLA-E alleles. These results can be explained by the small size of this retrospective monocentric cohort. Furthermore, reactivation of other viruses such as respiratory virus could be more relevant than CMV reactivation [36].

T cells are also able to recognize HLA-E binding peptides from bacteria such as* Mycobacterium tuberculosis*,* Salmonella*, and* Listeria* monocytogenes. In an unrelated allogenic stem cell transplantation study, homozygous HLA-E^*∗*^01:01 was identified as a risk for the occurrence of severe bacterial but not viral infections [16]. None of the HLA-E alleles were associated with a higher risk of bacterial infection at M1 and M3.

Another hypothesis is that HLA-E^*∗*^01:03 could lead to more efficient activation of CD8+ T cells alloreactivity. This mechanism has been suggested in a few related or unrelated stem cell allograft studies, showing that homozygous HLA-E^*∗*^01:03 induced a significant graft-versus-leukemia effect [17–19, 37]. In this context, HLA-E^*∗*^01:03 recipient may preferentially bind nonstandard minor histocompatibility antigen (mHag) peptides that can react with T cell activating receptors [18, 19, 37]. Thus, as for the GVL effect, this HLA-E dependent, alloreactive cellular process could specifically generate lung tissue inflammation. Furthermore, it has been shown that HLA-E exhibits alloantigenic properties that are indistinguishable from classical HLA class I molecules when expressed in HLA-E^*∗*^01:03 transgenic mice [24].

In summary, these data could be explained by the functional properties of the two HLA-E alleles in peptide affinity, cell surface expression, and potential lytic activity by NK cells or T cells. Therefore, a heterozygous status would offset the two homozygous states, each allele bringing benefits and risks to the overall survival of recipients via different mechanisms. For example, HLA-E^*∗*^01:03 could promote cellular alloreactivity mechanisms, triggered or not by viral infection via different receptors. In contrast, HLA-E^*∗*^01:01 could promote severe bacterial infection. Anyway, the impact of these two alleles in lung transplantation is concordant with the maintenance of these two alleles based on a balancing selection, meaning that there is a heterozygote advantage for individuals that are heterozygous at the HLA-E locus [38, 39].

Finally, the mechanism by which HLA-E alleles may promote CLAD and decrease long-term survival after LTx remains to be elucidated. The major limitation of this investigation is that it is a single-retrospective study and for certain analyses the patient cohort was small. It is possible that a few confounding factors not explored in this study may modify its interpretation. Furthermore, in the same way, a potential role of the donor's genotype and the possible interactions with the recipient's genotype could be studied. Thus, this association of HLA-E polymorphism with LTx occurrence needs verification using a larger cohort.

## Figures and Tables

**(a):** 

Variables	*p* value
Diseases other than cystic fibrosis	<0.001
HLA class II DSA	<0.001
HLA-G^*∗*^01:04~UTR3	0.001
Older recipients	0.001
CLAD occurrence	0.002
HLA class I DSA	0.03
HLA-B mismatch	0.03

**(b):** 

Variables	*p* value
HLA-DQ mismatch	0.002
HLA class I DSA	0.008
Diseases other than cystic fibrosis	0.01
HLA-DR mismatch	0.01
Single lung transplantation	0.03
HLA-G^*∗*^01:04~UTR3	0.03
Older recipients	0.05

**Table 2:** Baseline comparison of distribution and risk factors for 119 patients who underwent LTx according to HLA-E genotypes.

	E^*∗*^01:01/^*∗*^01:01	E^*∗*^01:01/^*∗*^01:03	E^*∗*^01:03/^*∗*^01:03	*p*	E^*∗*^01:01/^*∗*^01:01	E^*∗*^01:03 allele	*p*
Genotypes frequency (*N*, %)	38 (31.9)	57 (47.9)	24 (20.2)		38 (31.9)	81 (68.1)	
Male (*N*, %)	15 (40)	28 (49)	12 (50)	*0.60*	15 (39.5)	40 (49)	*0.33*
Age of recipient (SD)	39.1 (14.2)	40.23 (13)	39.6 (12)	*0.92*	39.1 (14.2)	40 (13)	*0.72*
Initial disease				*0.33*			*0.91*
Emphysema (*N*, %)	10 (26.3)	11 (19.3)	10 (41.7)		10 (26.3)	21 (25.9)	
Fibrosis (*N*, %)	8 (21.1)	8 (14)	5 (20.8)		8 (21.1)	13 (16.1)	
Cystic fibrosis (*N*, %)	14 (36.8)	28 (49.1%)	6 (25%)		14 (36.8)	34 (42.1)	
Others (*N*, %)	6 (15.8)	10 (17.5%)	3 (12.5)		6 (15.8)	13 (16)	
Transplant procedure				*0.58*			*0.92*
Lung and heart transplantation (*N*, %)	1 (2.6)	0 (0)	1 (4.2)		1 (2.6)	1 (1.2)	
Single lung transplantation (*N*, %)	8 (21.1)	14 (24.6)	4 (16.7)		8 (21.1)	18 (22.2)	
Bilateral lung transplantation (*N*, %)	29 (76.3)	43 (75.4)	19 (79.2)		29 (76.3)	62 (76.5)	
CMV status							
D+/R− (*N*, %)	6 (16.2)	7 (12.3)	4 (17.4)	*0.83*	6 (16.2)	11 (13.8)	*0.78*
D+/R+ or − (*N*, %)	19 (51)	31 (54)	9 (40)	*0.46*	19 (51)	40 (50)	*0.88*
D+ or −/R+ (*N*, %)	22 (58)	36 (63)	12 (50)	*0.54*	22 (58)	48 (59)	*0.88*
Mismatch HLA/6 (*N*)	5.03	5	4.83	*0.9*	5.03	4.95	*0.90*
Infection at M1 (*N*, %)	8 (53.3)	16 (47.1%)	2 (25)	*0.49*	8 (53.3)	18 (42.9)	*0.48*
Infection at M3 (*N*, %)	6 (46.2)	7 (38.9)	5 (50)	*0.83*	6 (46.2)	12 (42.9)	*0.84*
Infection in first year (*N*, %)	16 (42)	32 (56)	11 (45.8)	*0.37*	16 (42.1)	43 (53.1)	*0.26*
DSA at M1 (*N*, %)	7 (63.6)	13 (81.3)	5 (83.3)	*0.51*	7 (63.5)	18 (81.8)	*0.25*
DSA at M3 (*N*, %)	6 (66.7)	8 (66)	3 (60)	*1*	6 (66.7)	11 (64.7)	*0.92*
CLAD occurrence (*N*, %)	5 (22.7)	20 (40.8)	7 (58.3)	***0.06***	5 (22.7)	27 (44.3)	***0.02***
BOS (*N*, %)	5 (23.8)	16 (32.7)	6 (22.2)	*0.31*	5 (23.8)	22 (36.1)	***0.02***
RAS (*N*, %)	0 (0)	4 (8.2)	1 (8.3)	*0.41*	0 (0)	5 (8.2)	*0.32*
Overall survival (median, year)	2.37	3.7	2.6	***0.01***			

**Table 3:** Main risk factors for CLAD according to Cox regression multivariate analysis on recipient diagnosis, HLA-G^*∗*^01:04~UTR3 haplotype, and HLA-E^*∗*^01:03 allele (HR, hazard ratio; CI, confidence interval).

	Estimated HR (95% CI)	*p* value
Diagnosis		
Cystic fibrosis	Baseline	0.000
Emphysema	8.612 (3.113–23.824)	0.000
Fibrosis	1.515 (0.362–6.335)	0.569
Others	2.897 (0.822–10.214)	0.098
HLA-G^*∗*^01:04~UTR3	2.567 (0.889–7.412)	0.071
HLA-E^*∗*^01:03	3.563 (1.016–12.488)	0.047

## Abbreviations

LTx: Lung transplantation
CLAD: Chronic lung allograft dysfunction
FEV_1_: Forced Expiratory Volume measured during the first second
FVC: Forced vital capacity
DSA: Donor Specific Antibodies
BOS: Bronchiolitis obliterans syndrome
RAS: Restrictive allograft syndrome
CTL: Cytotoxic T-Lymphocytes
OS: Overall survival
DFS: Disease-free survival
ITIM: ImmunoTyrosine Inhibitor Module
CT: Computed Tomography
PFTs: Pulmonary function tests
RV: Residual volume
TLC: Total lung capacity
CMV: Cytomegalovirus
SD: Standard Deviation
HSCT: Hematopoietic Stem Cell Transplantation
TGF: Transforming Growth Factor
TLR: Toll-Like Receptor
IL: Interleukin
D: Donor
R: Recipient.
